# Response to chemotherapy in patients with recurrent rectal cancer in previously irradiated area

**DOI:** 10.1007/s00384-015-2270-2

**Published:** 2015-06-17

**Authors:** W. J. Alberda, B. C. Haberkorn, W. G. Morshuis, J. F. Oudendijk, J. J. Nuyttens, J. W. A. Burger, C. Verhoef, E. van Meerten

**Affiliations:** Department of Surgical Oncology, Erasmus MC Cancer Institute, Rotterdam, The Netherlands; Department of Medical Oncology, Erasmus MC Cancer Institute, P.O. Box 5201, 3008AE Rotterdam, The Netherlands; Department of Radiotherapy, Erasmus MC Cancer Institute, Rotterdam, The Netherlands

**Keywords:** Rectal neoplasms, Consolidation chemotherapy, Palliative care, Neoplasm metastasis

## Abstract

**Background:**

Tumor lesions in previously irradiated area may have a less favorable response to chemotherapy compared to tumor sites outside the radiation field. The aim of the present study was to evaluate the response to chemotherapy of locally recurrent rectal cancer (LRRC) within the previous radiation field compared to the response of distant metastases outside the radiation field.

**Patients and methods:**

All patients with LRRC referred between 2000 and 2012 to our tertiary university hospital were reviewed. The response to chemotherapy of LRRC within previously irradiated area was compared to the response of synchronous distant metastases outside the radiation field according to the Response Evaluation Criteria in Solid Tumors (RECIST).

**Results:**

Out of 363 cases with LRRC, 29 previously irradiated patients with distant metastases were treated with chemotherapy and eligible for analysis. Twenty-six patients (89 %) suffered a first recurrence and three patients (11 %) a second recurrence. These patients were followed with a median of 22 months (IQR, 9–40 months) and had a median survival of 33 months (IQR, 14–42). In 23 patients (79 %), the local recurrence showed stable disease, but the overall response rate of the local recurrences in the previously irradiated area was significantly lower than the response rate of distant metastases outside the radiation field (10 vs. 41 %,*p* = 0.034).

**Conclusions:**

Previously irradiated patients with LRRC have a lower response rate to chemotherapy of the local recurrence within the radiation field compared to the response rate of distant metastases outside the radiation field. This suggests that chemotherapy for local palliation may not have the desired effect.

## Introduction

Preoperative short-term radiotherapy (5 × 5 Gy) has evolved into an integrated part of the treatment of stage II and III rectal cancer in The Netherlands, because of the beneficial effect on local control [1].

Long-term radiotherapy (50 Gy) with or without concomitant chemotherapy has become standard of care in the treatment of locally advanced rectal cancer, because of improved local control and the effect of downsizing/downstaging, thereby facilitating the possibility of a complete surgical resection [2, 3]. Despite these advances, still 5–15 % of the patients develop a local recurrence [4]. The widespread use of neoadjuvant radiotherapy introduced a new problem: the treatment of locally recurrent rectal cancer (LRRC) in previously irradiated area.

The treatment of LRRC is a therapeutic challenge. Complete surgical resection is considered the only chance of durable local control and long-term survival [5, 6]. Unfortunately, only 31–40 % of the patients with LRRC have resectable disease [7, 8]. The majority is considered unresectable due to the presence of extensive synchronous distant metastases or an advanced local recurrence in which complete surgical resection is technically not feasible. These patients can only be offered palliative treatment, consisting of pelvic radiotherapy in case of pain or chemotherapy in case of metastasized disease.

The palliative treatment options in previously irradiated patients with LRRC are limited. Due to the previous radiotherapy, only a limited dose of radiation can be administered, and when treated with chemotherapy, the response of the local recurrence might be less favorable due to scarring and fibrosis of the pelvic tissue caused by the previous radiotherapy. This assumption is supported by a subgroup analysis of a meta-analysis, evaluating the response to chemotherapy for recurrent cervical cancer. Tumor recurrences within the previous radiation field showed a lower response rate to chemotherapy compared to the tumor recurrences outside the radiation field [9]. However, whether this also accounts for LRRC and the chemotherapeutic regimens used in this disease remains to be established.

The aim of the present study is to evaluate the response to chemotherapy of local recurrences in previously irradiated area compared to the response of distant metastases outside the radiation field within the same patient.

## Patients and methods

All patients with LRRC referred between January 2000 and December 2012 to the Erasmus MC Cancer Institute, a tertiary University hospital for the southwest region of The Netherlands, were analyzed. Patients were discussed in a multidisciplinary tumor board to determine the treatment strategy. At the time of diagnosis of LRRC, all patients were locally staged by a pelvic computed tomography scan (CT scan) or by magnetic resonance imaging (MRI) and were screened for distant metastases by a thoracic and abdominal imaging. LRRCs were diagnosed by histological biopsies or by imaging. The criteria for LRRC on imaging were as follows: a pelvic mass growing on consecutive imaging, a pelvic mass causing progressive ureter obstruction, or a pelvic mass with sacral or lateral pelvic bone invasion.

Previously irradiated patients who presented with a first or second local recurrence with synchronous distant metastases outside the radiation field were identified. Patients who were not considered candidates for LRRC surgery and were treated with chemotherapy were included for analysis. Patients receiving palliative re-irradiation for local pain relief prior to chemotherapeutic treatment were excluded, unless re-irradiation was administered at least 1 year before the start of the chemotherapeutic treatment and the local recurrence had grown in size on radiologic imaging. Data were collected from all referring hospitals and included demographics, radiotherapeutic reports, pathological reports, radiological imaging, and chemotherapeutic information.

Response to chemotherapy was assessed by two experienced medical oncologists and was scored according to Response Evaluation Criteria in Solid Tumors (RECIST) version 1.1 [10]. Tumor response was classified as a stable disease (SD), progressive disease (PD), partial response (PR), or complete response (CR). Overall response rate was defined as the sum of the patients with a PR or CR. Response evaluation was assessed after the first available follow-up CT scan after start of chemotherapy with a minimum of three and a maximum of nine completed courses of chemotherapy. Baseline CT scan had to be performed no more than 12 weeks before start of chemotherapy. Response evaluation of the local recurrence and the distant metastases was determined separately.

Statistical analysis was carried out using SPSS (version 20.0.0.1). Categorical data were reported as count (percentage) or median (interquartile range) as appropriate. Evaluation of distribution of response rates were performed by a chi-square test and a paired McNemar’s test. *p* values <0.05 were considered significant.

## Results

**Fig. 1:**
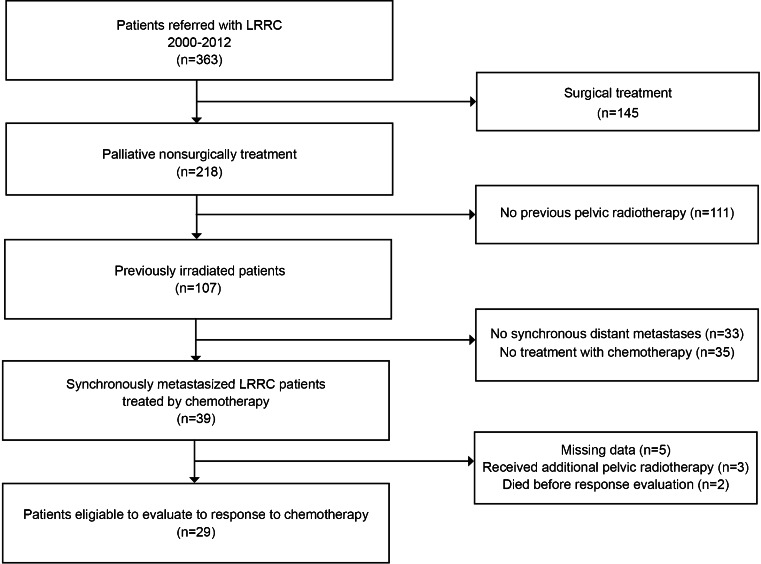
Flowchart of the patients referred to our hospital with LRRC and exclusion of LRRC patients

A total of 363 patients with LRRC were referred to our hospital; 218 patients (60 %) were not considered candidates for curative surgery and were offered palliative treatment. One hundred and seven patients received pelvic irradiation previously of which 74 had developed synchronous distant metastases outside the previous radiation field. Chemotherapy was administered to 39 patients. Ten patients were excluded due to missing data (*n* = 5), additional pelvic radiation within 1 year before the start of chemotherapy (*n* = 3), and death before tumor response evaluation (*n* = 2), leaving 29 patients evaluable for analysis (Fig. 1).

### Patient and tumor characteristics

Patients and tumor characteristics are outlined in Table 1. Twenty-six patients (89 %) suffered a first local recurrence, and three patients (11 %) suffered a second recurrence after LRRC surgery with curative intent. LRRC was histopathologically proven in 16 patients (55 %). The median interval between primary rectal surgery and LRRC diagnosis was 23 months (IQR, 15–36). Previous pelvic radiotherapy for the primary tumor or first local recurrence was a long course radiotherapy (44.6–50 Gy) in 12 patients (41 %), chemoradiotherapy (50 Gy) in 9 patients (28 %), and a short course radiotherapy (25 Gy) in 8 patients (28 %). The localization of the distant metastases was pulmonary in 16 patients (55 %), hepatic in 7 patients (24 %), both pulmonary and hepatic in 3 patients (10 %), and inguinal lymph nodes in 3 patients (10 %).

**Table 1:** Baseline patients and tumor characteristics

	Number of patients (%)
Total patients	29
Gender	
Male	22 (76)
Female	7 (24)
Age at diagnosis	65 (38–84)
Primary or LRRC surgery	
LAR	15 (52)
APR	11 (38)
Posterior exenteration	2 (7)
Total exenteration	1 (3)
Primary or LRRC resection margin	
R0	25 (86)
R1 (≤1 mm)	3 (10)
R2 (macroscopically incomplete)	1 (3)
Tumor stage	
T1–2	2 (7)
T3–4	27 (93)
Lymph node status	
N0	8 (28)
N+	21 (72)
Tumor differentiation	
Well	0
Moderate	17 (59)
Poor	5 (17)
Unknown	7 (24)

*LAR* low anterior resection, *APR* abdominoperineal resection

### Follow-up and response to chemotherapy

Patients were followed with a median of 22 months (IQR, 9–40). At last follow-up, five patients (17 %) were alive and 24 patients (82 %) died, resulting in a median survival of 33 months (IQR, 14–42). The used chemotherapeutic regimes are depicted in Table 2. Chemotherapy was administered after a median of 2 months (IQR, 1–8) after the diagnosis of LRRC, and response evaluation was done after a median of 3 cycles. The response rates to chemotherapy of the local recurrence and the distant metastases are outlined separately in Table 3. There was a significant difference between the overall response rate of local recurrence and distant metastases (10 vs. 41 %, *p* = 0.034). On individual basis, two patients with CR of the distant metastases had PR of the local recurrence. Of 10 patients with PR of the distant metastases, nine patients had SD and one had PD of the local recurrence. Of the 10 patients with SD of the distant metastases, eight patients had SD, one patient had PD, and one patient had PR of the local recurrence. Of the seven patients with PD of the distant metastases, six patients had SD and one patient had PD of the local recurrence. There was no significant difference in SD rate of the local recurrences of patients with histologically proven LRRC or radiologically detected LRRC (88 vs. 70 %, *p* = 0.36).

**Table 2:** Chemotherapeutic variables

	Number of patients (%)
Total patients	29
Number of cycles	6 (3–31)
Type of first-line chemotherapy	
Capecitabine	11
Capecitabine + oxaliplatin	6
Irinotecan	4
Fluorouracil + oxaliplatin + leucovorin	3
Capecitabine + oxaliplatin + bevacizumab	2
Cetuximab	1
Capecitabine + bevacizumab	1
Fluorouracil + leucovorin	1
Switch to second-line chemotherapy	
Yes	12
No	17

**Table 3:** Response to chemotherapy of the local recurrence and distant metastases

	Local recurrence (%)	Distant metastases (%)	*p* value
Response			
Complete response (CR)	–	2 (7)	–
Partial response (PR)	3 (10)	10 (35)	–
Stable disease (SD)	23 (79)	10 (35)	–
Progressive disease (PD)	3 (10)	7 (24)	0.006^a^
Overall response rate	3 (10)	12 (41)	0.034^b^

^a^Using chi-square

^b^Using McNemar’s test

## Discussion

The current study suggests a less favorable response rate (according to the RECIST) to chemotherapy of the local recurrence in previously irradiated area compared to the response rates of the distant metastases outside the radiation field within the same patient. The poor response rates of the local recurrences in previously irradiated area suggest that chemotherapeutic options may not have the desired effect for local palliation.

The response rate of the local recurrences in previously irradiated area was 10 %, whereas the 41 % response rate of the distant metastases was significantly higher. Although there is little data available about the response to chemotherapy of LRRC, the poor response is in line with studies evaluating the potential palliative effect of regional intra-arterial chemotherapy in LRRC. None of these studies were able to achieve an acceptable palliative result [11–14]. However, these studies were all conducted in the 1970s and 1980s before the introduction of the currently used chemotherapeutic regimens and did not solely included LRRC in previously irradiated area. Furthermore, the palliative results of these studies were based on subjective clinical symptoms and not on objective imaging. To our knowledge, this study is the first to assess the response of LRRC to contemporary chemotherapeutic regimens and evaluating the response of the local recurrences and distant metastases separately.

A possible explanation for the difference in response rate could be that previous radiotherapy and surgery alter the environment of the pelvis in which the local recurrence is located. Previous surgery may affect vascularization of the pelvic region, and radiotherapy leads to post-irradiation fibrosis and subsequently a reduced vascularization. This may prevent adequate local chemotherapeutic tissue levels, which are necessary to achieve tumor response. A comparable phenomenon was found in patients with recurrent cervical carcinoma. A pooled analysis of patients from multiple randomized controlled trials demonstrated a lower response rate to chemotherapy of tumor recurrences within the previous irradiated area compared to tumor recurrences outside the radiation field [9]. However, the analysis included studies comparing the response rates of patients with local recurrent disease after previous radiotherapy to the response rate patients who did not receive previous radiotherapy. Therefore, these results are more exposed to patient and tumor biology variability, which was minimalized in current study by comparing the response rate of the local recurrence and distant metastases within the same patient.

A second explanation for the difference in response rate may be that previous radiotherapy and surgery leads to a very fibrotic and rigid area, which makes the local recurrence within unable to shrink in contrast to the distant metastases outside the radiation and operation field. This may explain the remarkable high number of patients (79 %) with stable disease of the local recurrence, but not the finding that less patients had progressive disease of local recurrences in the previously irradiated area compared to the distant metastases outside the radiation field. This suggest that chemotherapy may have some influence on the local recurrence, but in comparison to the distant metastases, the response may be different due to genetic, biological, or environmental differences, whether or not caused by the radiotherapy.

The high rate of stable disease of the local recurrences might also be caused by the fact that not all LRRCs were histologically proven and that we simply evaluated non-malignant pelvic masses. However, both histologically proven and radiologically detected LRRCs showed a high rate of stable disease and we found no difference in stable disease rate of histologically proven and radiologically detected LRRCs.

Generally, the prognosis of patients with LRRC is poor. Moreover, previously irradiated patients with LRRC represent a group with even a poorer prognosis than “regular” not previously irradiated LRRC. This was demonstrated by an update of the Dutch TME trial. The vast majority of the patients who received radiotherapy for the primary tumor had distant metastases at diagnosis or developed them within the first 6 months after diagnosis. This resulted in a very poor median life expectancy of only 6 months [15]. In the current study, the survival rate of previously irradiated patients was significantly longer. Presumably, the patients in the current study are a selection of patients in generally good clinical condition and were therefore also considered candidates for chemotherapeutic treatment.

The main therapeutic problems of LRRC are the often disabling- and difficult to treat symptoms, such as severe pain and fistulating or bleeding tumors. The low response rate to chemotherapy as described in the current series clearly stresses the high need for novel treatment options and in particular for those patients with symptomatic local recurrences. Pelvic re-irradiation can provide pain relief in 65–83 % of the patients. Unfortunately, the duration of this pain relief is limited to a median of only 6–9 months and it can only be offered for a limited number of times [16–18]. Moreover, pelvic re-irradiation leaves distant metastases untreated and probably does not affect overall survival. A possible mechanism to improve the response to chemotherapy is to combine it with hyperthermia. Hyperthermia exposed parts of the body to high temperatures (42 °C), which causes increased intracellular drug uptake, enhanced DNA damage, and higher intra-tumor drug concentrations caused by an increased blood flow [19]. Future research should focus on combining hyperthermia and chemotherapy to investigate whether this approach improves the response rates of the local recurrences in previously irradiated area.

Chemotherapy is increasingly used in a potential curative preoperative setting for LRRC. Preoperative chemotherapy is administered to facilitate tumor downstaging and thus enhancing the chance of a complete resection. Complete resections are the most important prognostic factor for overall survival, and it is hypothesized achieving wider resection margins may improve outcome [6, 20]. The results of the current study contradict the potential downstaging effect of chemotherapy in previously irradiated patients. Therefore, the use of preoperative chemotherapy to induce tumor downstaging in previously irradiated patients needs further investigation.

Due to the retrospective nature of this analysis, this study has limitations. Moreover, there was no standard policy regarding the palliative treatment of patients with LRRC. Chemotherapy was only considered a suitable option in a small proportion of the patients with LRRC. This is illustrated by the fact that only 39 patients out of 74 LRRCs with synchronous distant metastases were treated with chemotherapy. This resulted in a relative small number of patients eligible for analysis. Furthermore, different chemotherapeutic regimens were used in the current study, which could lead to differences in response rate. However, this potential bias was ruled out by evaluating the response rate of distant metastases and local recurrence within the same individual patient.

In palliative treatment of LRRC, chemotherapy is administered to prolong survival and to achieve local symptom palliation. However, the current study did not evaluate the effect of chemotherapy on local symptom palliation, because evaluating local palliation is subjective and highly patient- and clinician dependent. Moreover, evaluating local palliation in a retrospective manner is highly unreliable. By using RECIST, we were able to evaluate response to chemotherapy in an objective manner.

In conclusion, previously irradiated patients with LRRC have a lower response rate to systemic chemotherapy of the local recurrence within the previous radiation field compared to the response rates of distant metastases outside the radiation field. This suggests that chemotherapeutic therapy for local palliation may not have the desired effect. Further studies are needed to improve treatment results, for example by combining chemotherapy with hyperthermia.
